# Bovine Leukemia Virus DNA in Human Breast Tissue

**DOI:** 10.3201/eid2005.131298

**Published:** 2014-05

**Authors:** Gertrude Case Buehring, Hua Min Shen, Hanne M. Jensen, K. Yeon Choi, Dejun Sun, Gerard Nuovo

**Affiliations:** University of California, Berkeley, California, USA (G.C. Buehring, H.M. Shen, K.Y. Choi, D. Sun);; University of California Davis Medical Center, Sacramento, California, USA (H.M. Jensen);; Ohio State University Comprehensive Cancer Center, Columbus, Ohio, USA (G. Nuovo)

**Keywords:** bovine leukemia virus, human, bovine, bovid, breast tissue, viruses, zoonosis, zoonoses, United States, breast cancer, leukemia, lymphoma, cancer, DNA

## Abstract

Bovine leukemia virus (BLV), a deltaretrovirus, causes B-cell leukemia/lymphoma in cattle and is prevalent in herds globally. A previous finding of antibodies against BLV in humans led us to examine the possibility of human infection with BLV. We focused on breast tissue because, in cattle, BLV DNA and protein have been found to be more abundant in mammary epithelium than in lymphocytes. In human breast tissue specimens, we identified BLV DNA by using nested liquid-phase PCR and DNA sequencing. Variations from the bovine reference sequence were infrequent and limited to base substitutions. In situ PCR and immunohistochemical testing localized BLV to the secretory epithelium of the breast. Our finding of BLV in human tissues indicates a risk for the acquisition and proliferation of this virus in humans. Further research is needed to determine whether BLV may play a direct role in human disease.

Bovine leukosis (B-cell leukemia/lymphoma), first described in 1871 in Lithuania, was believed to be an infectious disease because it spread through herds of cattle. In 1969, a virus isolated from cultured lymphocytes of cattle in an afflicted herd was identified as the agent of bovine leukosis (*1*). Since then, bovine leukemia virus (BLV) has been extensively investigated. It is a deltaretrovirus, closely related to human T-cell leukemia virus (HTLV) 1 (*2*), and has typical retroviral genome regions: *LTR* (long terminal repeat, promoter region); *gag* (group-specific antigen, capsid region); *pol* (polymerase, reverse transcription region, which synthesizes a DNA copy of the BLV RNA genome); and *env* (envelope). However, unlike other oncogenic retroviruses, deltaretroviruses have an additional region, *tax* (trans-activating region of the X gene), which has regulatory functions and is oncogenic to host cells. *tax* causes malignant transformation not through integration and insertional mutagenesis, as many retroviruses do, but by inhibition of DNA repair (base excision pathway) and trans-activating disruption of cellular growth control mechanisms (*2*).

BLV-infected cattle herds are found worldwide. In the United States, ≈38% of beef herds, 84% of all dairy herds, and 100% of large-scale dairy operation herds are infected (*3*,*4*). On average, clinical leukosis develops in <5% of these cattle, which are excluded from the market as a result (*1*), but BLV-infected lymphocytes are also found in the blood and milk of subclinically infected cows (*2*). Concerns that this virus might infect humans through exposure to food products from subclinically infected animals prompted 10 studies that used what were then (1975–1979) state-of-the-art immunologic methods to test serum samples from a collective total of 1,761 humans, including cancer patients, farm workers, and veterinarians (*5*). In these studies no antibodies against BLV were detected, prompting Burridge to conclude in his review article, “There is no epidemiological or serological evidence from human studies to indicate that BLV can infect man” (*5*). 

The advent of immunoblotting, ≈100 times more sensitive than techniques of the 1970s (*6*), enabled the detection of antibodies reactive with recombinant purified BLV p24 capsid protein in serum samples from 39% of 257 self-selected human volunteers (*7*). This study could not determine whether the antibodies were a response to infection or merely to heat-inactivated BLV consumed in food products. However, injection of sheep with raw milk from BLV-positive cows stimulated antibody production, whereas injection with pasteurized control milk did not (*8**,**9*). The finding of antibodies to BLV in humans prompted us to investigate human tissues for evidence of infection with BLV by using liquid-phase PCR (L-PCR), sequencing, in situ PCR, and immunohistochemical (IHC) testing. We focused on breast tissue because, in cattle, BLV DNA and p24 were detected in mammary tissue, whereas only BLV DNA was detected in lymphocytes (*10*).

## Materials and Methods

Cells and DNA used to test cross-reactivity of BLV primers and anti-BLV p24 monoclonal antibody (mAb) with other viruses are listed with their sources in Tables 1 and 2. Except for fetal lamb kidney (FLK) cells, cell lines were stored in liquid nitrogen until use. Before experiments were performed, the species of origin of all cell lines was authenticated by using the 2-pronged method (cytochrome oxidase housekeeping gene primers) (*11*).

**Table 1:** Lack of cross-reactivity of primers from 5 BLV genome regions with representatives of mammalian and avian retrovirus subfamilies and human exogenous and endogenous viruses previously identified in human breast tissue*

Virus	Subfamily	Cell line harboring virus (source†)	Nested liquid-phase PCR for BLV genome regions				
			*LTR*	*gag*	*pol*	*env*	*tax*
RSV	Alpharetrovirus	XC, rat cell line, transformed with RSV (CCL/NBRL)	–	–	–	–	–
MSV	Alpharetrovirus	F81, cat cell line, MSV infected (CCL/NBRL)	–	–	–	–	–
MMTV	Betaretrovirus	GR, mouse mammary tumor cell line (G. Firestone)	–	–	–	–	–
MPMV	Betaretrovirus	CMMT, rhesus monkey cell line (CCL/NBRL)	–	–	–	–	–
MuLV	Gammaretrovirus	JLSV5, mouse cell line (CCL/NBRL 14)	–	–	–	–	–
FeLV	Gammaretrovirus	FeLV 3281, cat cell line (CCL/NBRL)	–	–	–	–	–
BLV	Deltaretrovirus	FLK cell line (K. Radke)	+	+	+	+	+
		Bat_2_Clone_6_ cell line, BLV infected (K. Radke)	+	+	+	+	+
None		Tb_1_Lu, parental line of Bat_2_Clone_6_ before it was infected with BLV (K. Radke)	–	–	–	–	–
STLV	Deltaretrovirus	KIA, baboon cell line (ARRRP)	–	–	–	–	–
HTLV-1	Deltaretrovirus	MT2, human lymphocyte cell line (C. Hanson)	–	–	–	–	–
HTLV-2	Deltaretrovirus	Clone 19, human lymphocyte cell line (C. Hanson)	–	–	–	–	–
HIV-1	Lentivirus	H9, human cell line, HIV-1 infected (C. Hanson)	–	–	–	–	–
HIV-2	Lentivirus	H9, human cell line, HIV-2 infected (C. Hanson)	–	–	–	–	–
HPV-16	Papillomavirus	Caski, human uterine cervix cell line (ATCC)	–	–	–	–	–
HPV-18	Papillomavirus	HeLa, human uterine cervix cell line (CCL/NBRL)	–	–	–	–	–
EBV	Gamma-1 herpesvirus	Raji, human B-cell line (ATCC)	–	–	–	–	–
HERV-K	HERV, Class II	MCF-7, human breast cell line (ATCC)	–	–	–	–	–
		Purified, cloned HERV-K DNA (F. Wang-Johanning)	–	–	–	–	–

*In addition to assurances from the sources of the above biologicals, presence of the viruses in the respective cell lines was supported by rescue/syncytia formation when cell lines with replication defective viruses (RSV and MSV) were co-infected with replication competent retroviruses; positive reaction with primers specific for the virus (BLV, HTLV-1, -2, EBV, HPV-16); reaction with antibodies to the respective virus (MMTV, MMPV, MuLV); or receipt as formalin-fixed specimen so virus could not have been altered or lost (HIV-1, -2). BLV, bovine leukemia virus; *LTR*, long terminal repeat (promoter region); *gag*, group-specific antigen (capsid region); *pol*, polymerase (reverse transcription); *env*, envelope; *tax*, trans-activating region of the X gene; RSV, Rous sarcoma virus; MSV, murine sarcoma virus; MMTV, mouse mammary tumor virus; MPMV, Mason-Pfizer monkey virus; MuLV, murine leukemia virus; FeLV, feline leukemia virus; FLK, fetal lamb kidney; STLV, simian T-cell leukemia virus; HTLV, human T-cell leukemia virus; HPV, human papillomavirus; EBV, Epstein-Barr virus; HERV, human endogenous retrovirus. †Individually named sources listed in Acknowledgments. CCL/NBRL, former Cell Culture Laboratory of the Naval Bioscience Research Laboratory, Oakland, CA, USA; ARRRP, AIDS Research and Reference Reagent Program, National Institute of Allergy and Infectious Diseases (www.aidsreagent.org); ATCC, American Type Culture Collection.

**Table 2:** Lack of cross-reactivity of anti-BLV p24 (capsid region) mAb with common chronic viruses in human tissues*

Virus	Cells or tissue producing virus (source†)	Type of antibody to virus (source†)	Reaction of cells or tissue with	
			Ab to virus	mAb to BLV p24
BLV	FLK (positive control) (K. Radke)	mAb to BLV p24 (ARRRP no. 12145)	+	+
	Bat_2_Cl_6_ (positive control) (K. Radke)		+	+
None	Tb_1_Lu (negative control) (K. Radke)		–	–
MMTV	GR (G. Firestone)	pAb to MMTV (NCI-BCP)	+	–
HBV	Infected human liver sections (Dako)	pAb to HBV (Dako)	+	–
HTLV-1	MT-2 (C. Hanson)	pAb to HTLV-1 (ARRRP)	+	–
HTLV-2	Clone-19 (C. Hanson)	pAb to HTLV-2 (ARRRP)	+	–
HIV-1, -2	H9 HIV-1, -2 (C. Hanson)	pAb to HIV-1, -2 (ARRRP)	+	–
HPV	HPV-infected keratinocyte cultures (C. Meyers)	pAb to PV (Dako)	+	–
EBV	B95–8 (CCL/NBRL)	pAb to EBV (Dako)	+	–
CMV	Infected human lung sections (Dako)	pAb to CMV (Chemicon/Millipore)	+	–
HHV 1, 2	Infected human cells (Syva)	pAb to HHV 1, 2 (Chemicon/Millipore)	+	–

*BLV, bovine leukemia virus; mAb, monoclonal antibody; Ab, antibody; FLK, fetal lamb kidney; MMTV, mouse mammary tumor virus; pAb, polyclonal antibody; HBV, hepatitis B virus; HTLV, human T cell leukemia virus; HPV, human papillomavirus; PV, papillomavirus; EBV, Epstein-Barr virus; CMV, cytomegalovirus; HHV, human herpes virus. †Individually named sources listed in Acknowledgments. ARRRP, AIDS Research and Reference Reagent Program, National Institute of Allergy and Infectious Diseases (www.aidsreagent.org); NCI-BCP, National Cancer Institute, Breast Cancer Program; Dako, Glostrup, Denmark; CCL/NBRL, former Cell Culture Laboratory of the Naval Bioscience Research Laboratory, Oakland, CA, USA; Chemicon/Millipore, Ballerica, MA; Syva, Palo Alto, CA, USA.

Coded human samples were acquired from the Cooperative Human Tissue Network, a National Cancer Institute–supported tissue bank. Specimens were selected, without regard to patient age, race, or diagnosis, from archived breast tissues acquired from female patients who underwent breast surgery during 2000–2005 at participating hospitals in 4 catchments areas: Birmingham, Alabama; Pennsylvania; Ohio; and Oakland, California. The Institutional Review Board of the University of California, Berkeley (Berkeley, CA, USA) approved human subject use. Bovine control tissue came from the University of Wisconsin, Madison (Madison, WI, USA), as described (*10*).

PCR primers (Table 3) were examined for BLV specificity by using BLAST (http://blast.ncbi.nlm.nih.gov/Blast.cgi) to search for similar sequences in the nucleotide collection database, which includes exogenous viruses and human endogenous retroviruses such as HERV-K. The search was optimized for highly similar sequences.

**Table 3:** BLV primers and cycling conditions used for L-PCR, IS-PCR, and preparation of DNA for sequencing for detection of BLV in human breast tissue samples*

BLV gene	Primer pair sequences, 5′ → 3′†	Location in bp‡	Nested PCR role	Product length, bp	L-PCR/IS-PCR§	
					Annealing temperature, °C	Extension time, s
*LTR*	F: TAGGAGCCGCCACCGC	23–38	Outer	329	57/53	22/120
	R: GCGGTGGTCTCAGCCGA	352–336				
	F:AAACTGCAGCGTAAACCAGACAGAGACG	41–59	Inner	290	58/57	20/120
	R: CACCCTCCAAACCGTGCTTG	331–312				
*gag* (p24)	F: AACACTACGACTTGCAATCC	1068–1087	Outer	385	54/53	28/120
	R: GGTTCCTTAGGACTCCGTCG	1453–1434				
	F: ACCCTACTCCGGCTGACCTA	1097–1116	Inner	272	56/56	24/120
	R: CTTGGACGATGGTGGACCAA	1369–1350				
*pol*	F: TAGCCTACGTACATCTAACC	3238–3257	Outer	232	52/53	22/120
	R: AATCCAATTGTCTAGAGAGG	3470–3451				
	F: GGTCCACCCTGGTACTCTTC	3265–3284	Inner	157	57/56	18/120
	R: TATGGGCTTGGCATACGAGC	3422–3403				
*env*	F: TGATTGCGAGCCCCGATG	5144–5160	Outer	264	55/53	24/120
	R: TCTGACAGAGGGAACCCAGT	5408–5389				
	F: TGATTGCGAGCCCCGATG	5144–5160	Inner	230	55/56	22/120
	R: GGAAAGTCGGGTTGAGGG	5374–5357				
*tax*	F: CTTCGGGATCCATTACCTGA	7197–7216	Outer	373	55/55	26/120
	R: GCTCGAAGGGGGAAAGTGAA	7570–7551				
	F: ATGTCACCATCGATGCCTGG	7310–7329	Inner 1	113	55/53	15/120
	R: CATCGGCGGTCCAGTTGATA	7423–7404				
	F: GGCCCCACTCTCTACATGC	7265–7283	Inner 2 (sequencing)	206	56	22
	R: AGACATGCAGTCGAGGGAAC	7471–7452				

*BLV, bovine leukemia virus; L-PCR, nested liquid-phase PCR; IS-PCR, nested in situ PCR; *LTR*, long terminal repeat (promoter region); F, forward primer; R, reverse primer; *gag*, group-specific antigen (capsid region); *pol*, polymerase (reverse transcription); *env*, envelope; *tax*, trans-activating region of the X gene. †Reverse sequences are reversed and complementary to the proviral reference sequence. Primers were synthesized by Operon Biotechnologies, Huntsville, AL, USA. ‡Location according to reference sequence, GenBank accession no. EF600696. §For both rounds of nested liquid-phase PCR, cycling conditions were as follows: 1 cycle at 95°C for 2 min; 30 cycles at 95°C for 30 s, XX°C for 30 s, 72°C for XX; and 1 cycle at 72°C for 5 min, where XX represents annealing temperature and extension times, respectively, provided for each primer pair. For both rounds of nested in situ PCR, conditions were as follows: 1 cycle at 92°C for 10 min, 1 cycle at 91°C for 2 min, XX°C for 1.5 min; then 30 cycles of 91°C for 30 s, XX°C for 1.5 min, 72°C for 2 min; and 1 cycle of 72°C for 10 min, where XX represents IS-PCR annealing temperatures indicated for each primer pair.

For DNA extraction and quality control, cell lines were rinsed with Dulbecco phosphate-buffered saline (DPBS) and pelleted (500 × *g* for 3–5 min); DNA was then extracted by using the QIAamp DNA Mini Kit (QIAGEN, Hilden, Germany) according to the manufacturer’s cell protocol. DNA from human tissue specimens was extracted from frozen or deparaffinized formalin-fixed paraffin-embedded (FFPE) sections (5 µm thick) by using the QIAamp DNA Mini Kit according to the manufacturer’s tissue protocol. Overnight proteinase K digestion was extended 3–6 h to result in complete digestion, free of visible tissue particles. Extracted DNA quality was confirmed by amplification of a housekeeping gene sequence: human glyceraldehyde-3-phosphate dehydrogenase (GAPDH) for human, rhesus monkey, baboon, and bat material; murine GAPDH for mouse and rat cell lines; and bovine GAPDH for bovine, ovine, and feline cell lines (Table 4). Molecular contamination of extracted human DNA by BLV control DNA was monitored by using sheep-specific primers for the FLK cell line and plasmid vector primers for the C72/*tax*Neo line (bovine mammary epithelial cells stably transfected with BLV *tax* gene) (*12*), the only 2 BLV-containing cell lines previously grown in the laboratory in which testing was conducted. 

**Table 4:** Primers and reaction conditions for GAPDH gene amplifications used to verify quality of BLV DNA extracted from human breast tissue samples, human cell lines, and animal cell lines*

Gene	Primer pair sequences, 5′ → 3′†	Location in bp‡	Product length, bp	Annealing temperature, °C§	Extension time, s§
Human GAPDH	F: GAGTCAACGGATTTGGTCGT	194–213	237	50	22
	R: TTGATTTTGGAGGGATCTCG	431–412			
Mouse GAPDH	F: AGCTTGTCATCAACGGGAAG	246–265	796	58	60
	R: ATGTAGGCCATGAGGTCCAC	1041–1022			
Bovine GAPDH	F: CCTTCATTGACCTTCACTACATGGTCTA	172–199	857	59	60
	R: GCTGTAGCCAAATTCATTGTCGTACCA	1028–1002			

*GAPDH, glyceraldehyde-3-phosphate dehydrogenase; BLV, bovine leukemia virus; F, forward primer; R, reverse primer. †Reverse sequences are reversed and complementary to the published genomic sequences. Primers were synthesized by Operon Biotechnologies, Huntsville, AL, USA. ‡Sequence bp numbering according to GenBank no. NM 002046.4 (human), XM 001476707.3 (mouse), and NM 001034034.2 (bovine).  §Cycling conditions were 1 cycle at 95°C for 2 min; 35 cycles at 95°C for 30 s; XX°C for 30 s, 72°C for XX sec; and 1 cycle at 72°C for 5 min, where XX represents annealing temperature and extension times, respectively, for primer pairs.

For nested L-PCR, extracted DNA (0.85 µg) was added to 50 μL of PCR mix (2.0 mmol/L MgCl_2_, 0.2 mmol/L dNTPs, 0.025 U/µL Taq polymerase [all from Promega, Madison, WI, USA], and 0.2 μmol/L outer primers for each BLV gene [Table 3]) in Hot Start Micro 50 tubes (MolecularBio Products, San Diego, CA, USA). For the second (nested) round of amplification, 2 μL of the first-round PCR product was added to a new tube of the same reaction mix with inner primers for the corresponding genome region (Table 3). Cycling conditions are given in Table 3. Reaction mix and conditions, including MgCl_2_ concentrations, were optimized by using the BLV-positive FLK fibroblasts and BLV-negative bat lung fibroblasts (Tb_1_Lu). Sensitivity of the nested L-PCR was determined by using the housekeeping gene GAPDH, which occurs in humans as 1 copy/cell (*13*). DNA was extracted from 1 million cells of the human cell line MCF7 (Table 1) and serially diluted to an end point equivalent of 1 cell. Nested L-PCR performed on each dilution established the sensitivity at ≤10 copies.

L-PCR, which requires initial tissue digestion to extract DNA, is prone to molecular contamination. We reduced this probability by separating PCR procedure steps into 3 locations: 1) preparation of PCR mixes in a dedicated, locked, DNA-free room with entrance and ventilation system separate from the main laboratory and with dedicated laminar flow hood, small equipment, plasticware, and reagents; 2) addition of template DNA in a different room in a small hood dedicated to specimen handling, with DNA easily decontaminated by UV light and DNA decontamination solution (RNase AWAY; Molecular BioProducts, San Diego, CA, USA) applied to equipment and surfaces before and after handling each specimen; and 3) processing and addition of positive control samples last, and only in a fume hood (velocity = 127 fpm) vented to atmospheric air, thus preventing contaminating aerosols from entering the general work area.

For sequencing, BLV amplicons obtained by nested L-PCR were separated by electrophoreses in a 1% agarose gel at 100 V, excised from the gel, and cleaned by using the Zymoclean Gel DNA Recovery Kit (Zymo Research, Irvine, CA, USA). The requested sample of 100 ng of DNA in 12 µL water and 1 μL of the 5 μmol/L sequencing primer stock solution of the inner primer pair for each genome region (Table 3) was submitted to the University of California, Berkeley, DNA Sequencing Facility for direct sequencing. For *tax*, inner primer 2 was used to obtain a longer product. DNA for sequencing was obtained from 2–5 amplifications and was sequenced at least once in forward (5′) and reverse (3′) directions. Sequences were checked against corresponding electropherograms. Variations from the reference sequence (GenBank accession no. EF600696) were considered valid only if they matched in both forward and reverse directions. Targeted sequences were relatively short because formalin causes DNA breaks, making it difficult to obtain sequences of >130 nt (*14*).

Nested in situ PCR (IS-PCR), adapted from Nuovo (*15*), was used to identify which cell types within tissues were BLV positive. Thick suspensions of detached, rinsed control cells were smeared on enhanced adherence glass microscope slides, air dried, and fixed for 16–18 h with 10% buffered neutral formalin. To enhance entry of PCR mix into cells, samples were made permeable by digestion with 2 mg/mL pepsin in 0.1 N HCl (40–80 min for tissue sections; 20 min for control cell smears), pepsin inactivation solution (100 mmol/L Tris-HCl, 100 mmol/L NaCl, pH 7.4) applied for 1 min, followed by a DPBS rinse and 5 min in absolute ethanol. Samples, run in duplicate, were surrounded with a 15 × 15 mm frame seal chamber (Bio-Rad, Hercules, CA, USA); 60 μL of PCR mix were then placed into the chamber, and the plastic cover was sealed over the frame. The PCR mixture was 4.0 mmol/L MgCl_2_, 0.4 mmol/L dNTPs, 1 μmol/L primers (Operon Biotechnologies, Huntsville, AL, USA), 0.06% bovine serum albumin, 8 μmol/L digoxigenin-11-dUTP (dig) (Hoffman-La Roche, Basel, Switzerland), and 0.053 U/μL Amplitaq Gold DNA Polymerase (Applied Biosystems, Foster City, CA, USA), a Taq polymerase activated only at ≥92°C, designed to reduce false positives from nonspecific DNA repair by Taq at cooler temperatures (*15*). Primers were the same as those used for L-PCR (Table 3). Slides were placed into an IS-PCR machine (Hybaid Thermo OmniSlide; Cambridge Biosystems, Cambridge, UK) for amplification. After each round, covers and chambers were removed, and slides were rinsed in DPBS. After the second round, endogenous peroxidase was quenched 30 min in 3% H_2_O_2_ in methanol. Label incorporated into PCR products was detected by anti-dig antibodies in an avidin-biotin-immunoperoxidase reaction (Hoffman-La Roche). The chromagen was diaminobenzidine. Outcome measurement was a semiquantitative judgment of color density of cells: 1+, light tan; 2+, medium tan; 3+, dark brown; 4+, almost black. Ratings of ≥2+ were considered positive. No donor information or results of L-PCR were available at the time of slide evaluation.

Initially, the BLV-positive control cell line (FLK) and the BLV-negative cell line (Tb_1_Lu) were used to optimize the reaction and ensure no false-positive reaction in the negative cell line. Control testing was run simultaneously with each batch of human tissue assays: 1) positive control, a smear of BLV-positive cells (FLK cell line) reacted with complete PCR mix; 2) negative controls, a smear of FLK cells and an adjacent serial section of each specimen reacted with PCR mix minus primers, to rule out false-positive reactions unique to each tissue resulting from unquenched endogenous peroxidase, nonspecific reaction of the sheep antibodies used in the final immunoperoxidase detection, or nonspecific DNA repair by Taq polymerase; 3) permeabilization control for entry of PCR mix into cells, with an adjacent serial section of each tissue reacted with PCR mix different from that for IS-PCR by omission of primers, 4.5 mmol/L concentration for MgCl_2_, and use of a different Taq polymerase (Promega) that reacts nonspecifically at cooler temperatures (4°C–50°C) to repair DNA. Specimens were scored positive only if mammary epithelial cells were positive and the background control slide (adjacent section) without primers was negative for the corresponding area of mammary epithelial cells. Specimens were scored negative only if the sample and its background control section were negative and the permeabilized control exhibited dig incorporation into cell nuclei resulting from Taq polymerase DNA repair, confirming entry of PCR mix into cells (*15*).

IHC testing for BLV p24 was performed by using formalin-fixed cell smears and deparaffinized FFPE tissue sections (5 μm) on superadherent microscope slides. Samples were quenched of endogenous peroxidase for 30 min in 3% H_2_O_2_ in methanol and rinsed in DPBS; antigens were then unmasked by incubation in citrate buffer (0.1 mmol/L sodium citrate, 0.04 mmol/L citric acid, pH 6.0) for 25 min at 95°C in individual plastic containers to prevent cross-transfer of tissue material. Unmasking was followed by a DPBS rinse, and an avidin-biotin-immunoperoxidase procedure (Vectastain Elite ABC Kit; Vector Laboratories, Burlingame, CA, USA) was performed according to the manufacturer’s instructions. Blocking serum was 1.5% fetal horse serum in DPBS. Primary antibody in blocking serum (1:10) was a hybridoma-produced mouse mAb against BLV p24 (AIDS Research and Reference Reagent Program, National Institute of Allergy and Infectious Diseases; www.aidsreagent.org) (*16*); specificity was validated by immunoblot reactivity with purified recombinant p24 (*7*). Secondary antibody was 1.5% horse anti-mouse IgG (Vector) in blocking serum. The chromagen was diaminobenzidine. Negative controls were the BLV-negative control cell line Tb1Lu and adjacent human tissue sections reacted with fresh hybridoma medium (diluted 1:10 in blocking buffer) substituted for primary antibody. The positive control was FLK, which is known to replicate BLV. Outcome measurement was a semiquantitative judgment of color density, as described for IS-PCR.

## Results

**Figure 1:**
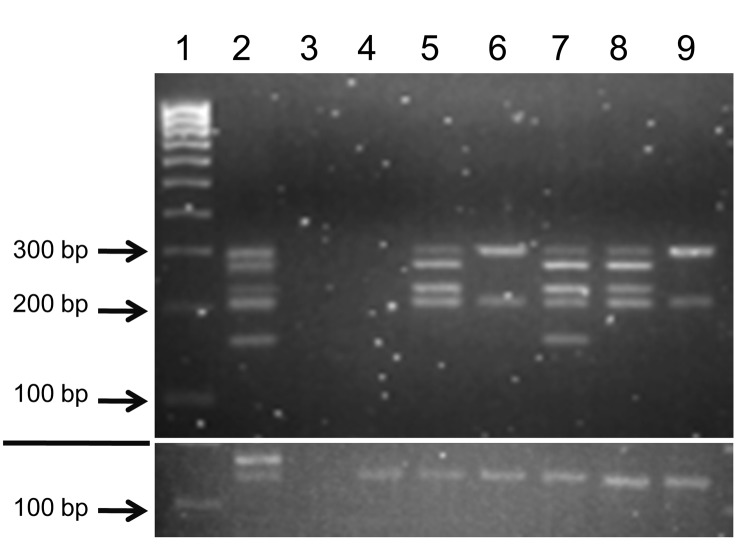
Amplification of bovine leukemia virus (BLV) genome regions in human breast tissue specimens. Nested liquid-phase PCR, using primers from 5 BLV genome regions, was used to amplify products from DNA extracted from breast tissues of 6 human donors. PCR products for each tissue were loaded into 1 well and separated by agarose gel (3.5%) electrophoresis on the basis of size differences: long terminal repeat, 290 bp; group-specific antigen, 272 bp; envelope, 230 bp; trans-activating gene of the X region, 206 bp; polymerase, 157 bp. The section below the white line shows the glyceraldehyde 3-phosphate dehydrogenase amplification of each sample as an indicator of DNA quality. Lane 1, molecular weight marker (HyperLadder IV; Bioline, Taunton, MA, USA); lane 2, fetal lamb kidney cell line, positive control; lane 3, no-template-DNA negative control (water substituted for DNA template); lane 4, human sample 143; lane 5, human sample 236; lane 6, human sample 010; lane 7, human sample 20874; lane 8, human sample 23803; lane 9, human sample 0253.

**Figure 2:**
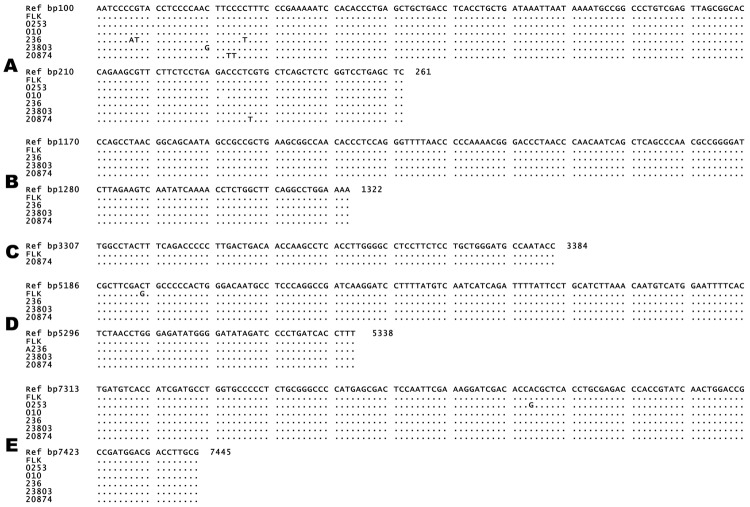
Partial sequences of 5 genome regions of bovine leukemia virus (BLV) DNA isolated from human breast tissue samples: A) long terminal repeat; B) group-specific antigen; C) polymerase; D) envelope; E) trans-activating gene of the X region. Reference (Ref) sequences (GenBank accession no. EF600696) are shown; dots indicate where samples have no difference from the reference sequence. Fetal lamb kidney (FLK) is shown as positive control cell line. Human samples are listed by sample number. Sequences displayed are shorter than the amplicon each primer set amplified because only overlapping regions of both forward and reverse sequencing are shown. Missing sequences indicate that the sample did not test positive for those genome regions.

Human samples were selected from 219 FFPE breast tissue samples; 97 (44%) samples had positive results for BLV by nested IS-PCR that used primers from the most conserved BLV genome region, *tax*. Five *tax-*positive samples and 1 *tax-*negative sample (as a control) were chosen for in-depth molecular analysis on the basis of BLV status and sample size large enough and with enough mammary epithelial cells to yield sufficient material for multiple assays and extensive quality control tests. BLV was detected in DNA extracted from human tissues by using nested L-PCR. All 5 *tax-*positive samples chosen were positive for the *LTR* region but showed varying results for other BLV genome regions (Table 5; Figure 1). Sequences of all samples positive for BLV had high identity (E value <1.2) only to BLV nucleotide sequences deposited in GenBank, which suggests that these isolates did not represent some other entity. Variations from the BLV reference sequence were infrequent, and all involved base substitutions (Figure 2).

**Table 5:** PCR results for detection of BLV in breast tissue samples from 6 women*

Sample code	Sample pathology	Patient age, y	BLV genome regions						
			L-PCR						IS-PCR
			*LTR*	*gag*	*pol*	*env*	*tax*		*tax*
143	Malignant	63	–	–	–	–	–		–
0253	Malignant	47	+	–	–	–	+		+
010	Malignant	48	+	–	–	–	+		+
236	Nonmalignant	54	+	+	–	+	+		+
23803	Nonmalignant	50	+	+	–	+	+		+
20874	Nonmalignant	53	+	+	+	+	+		+

*BLV, bovine leukemia virus; L-PCR, nested liquid-phase PCR; IS-PCR, nested in situ PCR; *LTR*, long terminal repeat (promoter region); *gag*, group-specific antigen (capsid region); *pol*, polymerase (reverse transcription); *env*, envelope; *tax*, transactivating region of the X gene.

**Figure 3:**
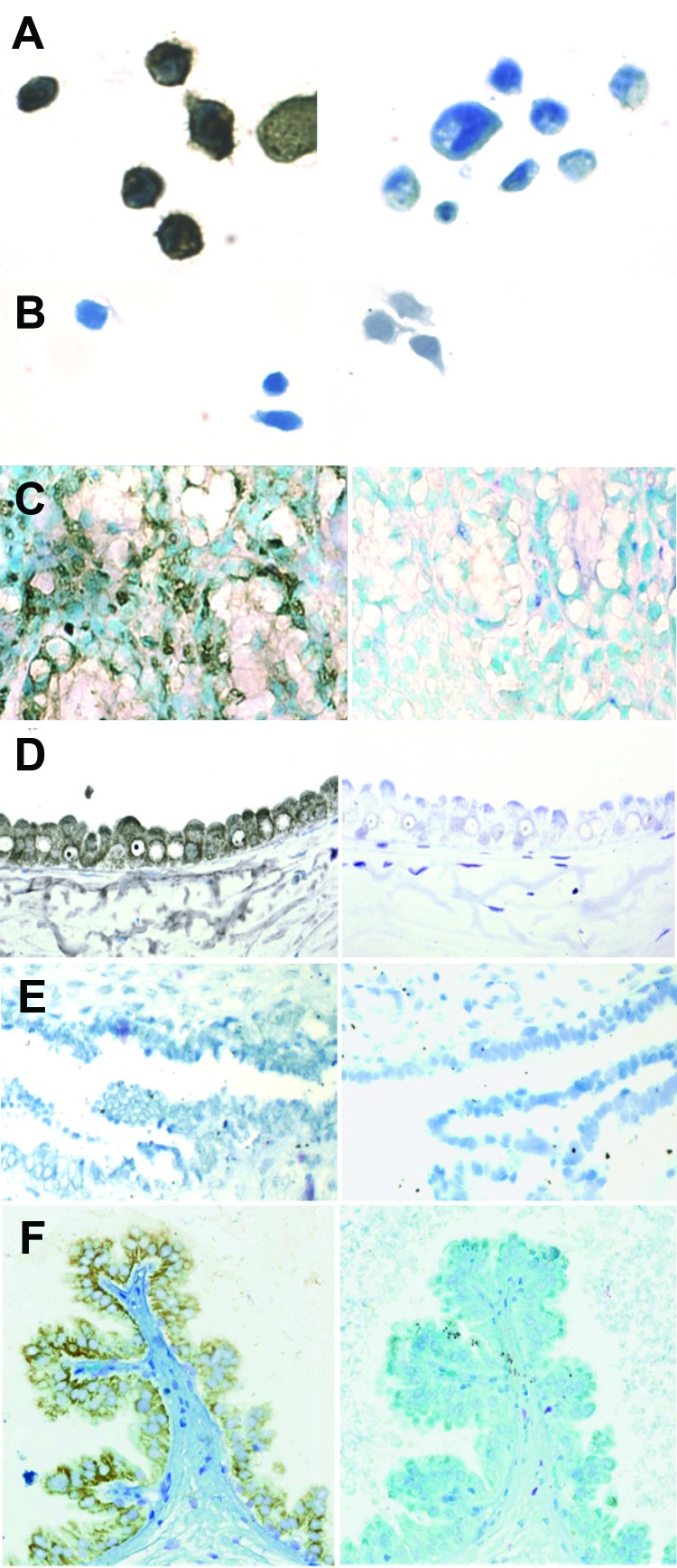
Localization of bovine leukemia virus (BLV) in human breast tissue and bovine mammary epithelium samples detected by in situ PCR for the BLV *tax* region and immunohistochemical testing for p24 capsid protein. A) BLV-positive fetal lamb kidney (FLK) cell line. Brown at left indicates positive diaminobenzidine endpoint immunoperoxidase reaction to detect digoxygenin incorporated into PCR product within FLK cells. FLK cells reacted with PCR reaction mix without primers (right) to check for false-positive background show no reaction. Original magnification ×400. B) BLV-negative cell line Tb_1_Lu with (left) and without (right) primers. No reaction occurred with either condition because the cell line has no BLV to amplify and shows no nonspecific background. Original magnification ×400. C) BLV-positive lactating bovine mammary gland tissue with (left) and without (right) *tax* primers in the PCR mix. Dark brown at left indicates positive cells, some surrounding lumens filled with milk. Lack of reactive cells in sample at right without primers indicates reaction was not a false positive due to nonspecific factors inherent in the tissue. Original magnification ×100. D) BLV-positive human tissue sample 010 reacted with *tax* primers. Dark brown at left indicates epithelial cells facing the lumen of a large cyst. Lack of reactive cells in sample at right without primers indicates reaction was not a false positive. Original magnification ×100. E) BLV-negative human tissue sample 143 exposed to PCR mix with (left) and without (right) primers showing no reaction with either condition in the epithelium of the long duct. Original magnification ×40. F) BLV-positive human tissue reacted with monoclonal antibody to BLV p24 (left) in an avidin-biotin-immunoperoxidase assay. Brown indicates end-point reaction in cytoplasm of epithelium projecting into the cyst lumen on a stalk of collagenous stroma. Note lack of reaction in sample at right with hybridoma medium substituted for primary antibody. Original magnification ×40. All cells and tissues were counterstained with Diff-Quik Solution II (Dade Behring, Newark, DE, USA).

Nested IS-PCR was used to identify the cell type in which the PCR product was localized. Figure 3 shows IS-PCR results obtained by using *tax* primers for the BLV-negative human sample (no. 143), 1 of the BLV-positive samples (no. 010), the positive and negative cell line controls, and a BLV *tax*-positive bovine mammary gland sample. The site of the amplified BLV DNA was the secretory mammary epithelium, identified by an anatomic pathologist (H.M.J.). Table 5 summarizes the results for L-PCR and IS-PCR (*tax*) testing of the 6 tissue samples that were studied in depth.

Validation of the IS-PCR results for a subset of 7 samples (3 *tax* negative and 4 *tax* positive) was performed by an independent laboratory by using control cell smears and coded FFPE sections sent from our laboratory with no information about the human patients, tissue pathology, or our results. The detection method was PCR in situ hybridization, in which the PCR occurs in situ but with no label incorporated during amplification (*15*). Labeled probes specific for the BLV *tax* region were applied after amplification. The independent laboratory confirmed with 100% concordance the results we obtained by using nested IS-PCR and L-PCR for the *tax* genome region (Table 6).

**Table 6:** Validation of PCR results for representative breast tissues tested at the University of California Berkeley, conducted at an independent laboratory*

Sample code	Sample pathology	IS-PCR result	PCR in situ hybridization result†
B702	Nonmalignant	–	–
143‡	Malignant	–	–
B984	Malignant	–	–
236‡	Nonmalignant	+	+
B975	Nonmalignant	+	+
154	Malignant	+	+
253‡	Malignant	+	+

*IS-PCR, nested in situ PCR. †Ohio State University Comprehensive Cancer Center, Columbus, OH, USA. ‡Samples were among those used for in-depth study reported in this article (Table 5).

Of the 215 human breast tissue samples tested by IHC, 12 (6%) had positive results for BLV p24. The reaction was confined to secretory mammary epithelium, and distribution of both BLV DNA and p24 in mammary epithelial cells was duct and lobule specific; that is, virtually all cells in the duct or lobule would be BLV positive but neighboring ducts or lobules could be completely negative. A BLV p24–positive breast tissue sample is shown in Figure 3, panel F.

**Figure 4:**
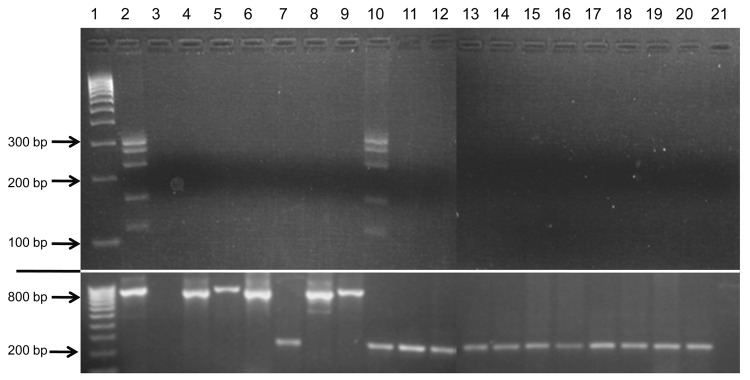
Test results showing lack of cross**-**reactivity of bovine leukemia virus (BLV)–specific primers with representatives of all mammalian and avian retrovirus subfamilies and human exogenous and endogenous viruses previously identified in human breast tissue. Nested liquid-phase PCR used primers from 5 BLV genome regions with template DNA from the viruses in lanes 4–10 and 12–21. PCR products for each virus, loaded into 1 well, were separated by agarose gel (1.5%) electrophoresis on the basis of size differences. Amplicons were generated only for known BLV-positive cell lines (FLK and Bat_2_Cl_6_). Samples in lanes 13–21 were run simultaneously in the same gel in wells below samples in lanes 4–12. The section below the white line shows glyceraldehyde 3-phosphate dehydrogenase (GAPDH) amplification of each sample to indicate DNA quality. Human GAPDH primers were used for human, rhesus monkey, baboon, and bat cell lines (amplicon = 237 bp); murine GAPDH for mouse and rat cell lines (796 bp); and bovine GAPDH for bovine, ovine, and feline cell lines (857 bp). Lane 1, molecular weight marker (HyperLadder IV; Bioline, Taunton, MA, USA), lane 2, fetal lamb kidney cell line, positive control; lane 3, water substituted for DNA template, negative control; lane 4, Rous sarcoma virus; lane 5, murine sarcoma virus; lane 6, mouse mammary tumor virus; lane 7, Mason-Pfizer monkey virus; lane 8, murine leukemia virus; lane 9, feline leukemia virus; lane 10, BLV Bat_2_Cl_6_; lane 11, Tb_1_Lu (known BLV-negative cell line), negative control; lane 12, simian T-cell leukemia virus; lane 13, human T-cell leukemia virus 1; lane 14, human T-cell leukemia virus 2; lane 15, HIV-1; lane 16, HIV-2; lane 17, human papillomavirus 16; lane 18, human papillomavirus 18; lane 19, Epstein-Barr virus; lane 20, human endogenous retrovirus K; lane 21, *env* of human endogenous retrovirus K.

GenBank searches indicated complete identity (E value ≤1.1) of PCR primers (Table 3) only with BLV sequences. L-PCR further substantiated primer specificity (Figure 4; Table 1) by demonstrating no cross-reactivity (amplicon generation) by using template DNA from cell lines harboring representatives of all mammalian and avian oncogenic retroviral subfamilies and human lentiviruses; viruses previously reported in human breast tissues (Epstein-Barr virus, human papillomavirus, mouse mammary tumor virus–like sequences) (*17*); and human endogenous retrovirus HERV-K (Table 1). Extracted DNA met quality control standards of exhibiting a housekeeping gene (GAPDH; Figure 4). mAb specificity was supported by lack of cross-reactivity with cell lines or by tissues replicating viruses previously identified in human breast tissues (Epstein-Barr virus, human papillomavirus, and mouse mammary tumor virus–like sequences) (*17*) or that cause chronic human infections (hepatitis B virus, human T-cell leukemia viruses 1 and 2, HIV 1 and 2, human herpesviruses 1 and 2, and cytomegalovirus) (Table 2).

Several methods were used to test for cell and molecular contamination. The no-template-DNA control run with each assay ruled out contamination in commercial DNA isolation columns and PCR reagents; in addition, contamination of human DNA samples by FLK DNA was ruled out by testing human DNA with sheep-specific primers (*11*), and contamination of FLK with other cell lines, including human, was ruled out by using species-specific primers (*11*). Sequences from the human isolates also provided strong evidence against contamination; none were exact matches with each other or with any Genbank BLV sequence, which includes the FLK-positive control cell line and bovine specimens previously sequenced in our laboratory. Furthermore, the FLK stock cell line has a signature base substitution (bp 5194; GenBank accession no. EF600696) in the *env* region; this substitution is unique among sequences deposited in GenBank and was not detected in the human BLV isolates we investigated (Figure 2). The positive results obtained by using in situ methods (IHC and IS-PCR) demonstrating the signal within individual formalin-fixed mammary epithelial cells are further evidence that the positive reactions do not represent contamination of PCR mix. Previous reports have documented that contaminating DNA cannot cause a false-positive reaction with PCR in situ hybridization in FFPE tissues (*15*). Negative controls (adjacent tissue sections without BLV-specific primers or antibody) run with each in situ assay were all negative, which suggests that positive signals were not false positives resulting from nonspecific reactions.

## Discussion

During the 4 decades since BLV was identified in cattle, there has been considerable interest in determining whether humans could become infected with BLV, especially because cattle are a major food source. As in early serologic studies, previous cellular and molecular studies reported no evidence of human infection with BLV. An explanation for some of the negative findings (*18**–**22*) could be that sufficiently sensitive reagents and techniques such as PCR and sequencing were not available when the studies were conducted. Most previous studies also focused on leukocytes, the cell type involved in bovine leukemia/lymphoma (*18**–**27*). We focused instead on mammary epithelium, in which we had detected BLV DNA and protein in cattle (*10*). In situ techniques (i.e., IS-PCR, PCR in situ hybridization, and IHC) enabled confirmation that BLV was localized within mammary epithelial cells.

Evidence for BLV DNA and protein in humans is not surprising. Many viruses, including those that are oncogenic, are known to cross species naturally, and most microbial species pathogenic in humans are speculated to have had a animal origin at some point in human evolution. Once in the human population, most zoonotic viruses can be transmitted among humans, a process that poses the most serious threat to human health (*28*). BLV is known to cross species readily; the virus infects capybara, zebus, and water buffaloes naturally and sheep, goats, pigs, rabbits, rats, and chickens experimentally (*1*). Human cells (fibroblasts) are susceptible to infection with BLV in vitro (*29*).

The lack of *gag, pol*, and *env* sequences in some of the BLV-positive panel samples and the presence of *LTR* and *tax* sequences in all of them is consistent with results reported for the closely related HTLV-1 (*30*). Deletions in *gag, pol,* and *env* were observed in HTLV-1 isolates from 25.7%–56% of adult T-cell leukemia patients, and frequency increased with clinical progression of leukemia (*30*). Such deletions are postulated to be advantageous to the virus by enabling escape from immune surveillance. In contrast, the *LTR* and *tax* regions of HTLV-1 are highly conserved. Analogous results were observed in specimens from cattle infected with BLV; deletions involving parts of *gag* and *env* and all of *pol* were frequent (*2*).

Overall, the human BLV isolates differed from the reference sequence by only a few base substitutions, a finding that fits well with the biology of deltaretroviruses, which have infrequent interhost transmission and remain largely latent within the host, probably as a strategy to escape the host’s immune response (*31*). Infectivity of deltaretroviruses occurs primarily by cell–cell contact, not by extracellular virions (*32*), and BLV virions have not been found in the peripheral blood of infected cattle (*2*). In our study, evidence of the capsid protein p24 was infrequent (12/215 [6%]) but consistent with the concept that, in some humans, BLV could be replicating.

Mutation rate tends to be lower for latent viruses than for viruses that escape immune attack by rapidly mutating to outpace the host’s immune response (e.g., HIV [*2**,**31*]). Four of the 7 *LTR* base substitutions we observed (in 3 human BLV isolates) occurred in the PU box, a short (12 bp) protein-binding region that, when mutated, decreases basal gene expression (*2**,**33*). This expression would facilitate evasion of the host’s immune response and would thus be likely to be selected for during virus evolution. Transcription in BLV-infected bovine lymphocytes is rare, identified in only ≈1/50,000 peripheral blood cells (*2*).

In summary, multiple lines of evidence support the conclusion that the BLV DNA and protein we found are more likely to represent the in vivo presence of BLV in humans than to represent some other virus, molecular laboratory contamination, or an artifactual nonspecific reaction. In view of the potential public health implications of BLV in humans, future research should address how humans acquire BLV infection, how frequently BLV infection occurs in different populations, and whether the virus is associated with human disease. 
